# Post-stroke rehabilitation of the ankle joint with a low cost monoarticular ankle robotic exoskeleton: Preliminary results

**DOI:** 10.3389/fbioe.2022.1015201

**Published:** 2022-11-25

**Authors:** Guillermo Asín-Prieto, Silvana Mercante, Raúl Rojas, Mariangeles Navas, Daiana Gomez, Melisa Toledo, Aitor Martínez-Expósito, Juan C. Moreno

**Affiliations:** ^1^ Neural Rehabilitation Group, Cajal Institute, CSIC—Spanish National Research Council, Madrid, Spain; ^2^ J. N. Lencinas Hospital, Mendoza, Argentina; ^3^ Department of Anatomy, Histology and Neuroscience, Universidad Autónoma de Madrid, Madrid, Spain

**Keywords:** hemiplegia, rehabilitation, robotics, gait, ankle

## Abstract

**Introduction:** Stroke generates a high rate of disability and, in particular, ankle spasticity is a sequelae that interferes with the execution of daily activities. Robotic devices have been proposed to offer rehabilitation treatments to recover control of ankle muscles and hence to improve gait function.

**Objective:** The aim of this study is to investigate the effects of passive stretching, combined with active and resisted movement, accompanied by visual feedback, by means of playful interactive software using a low-cost monoarticular robot (MEXO) in patients with stroke sequelae and spastic ankle.

**Methods:** An open, uncontrolled, non–randomised, quasi–experimental study of 6 weeks duration has been completed. A protocol has been defined to determine the usability, safety and potential benefits of supplementary treatment with the MEXO interactive system in a group of patients. Nine volunteer patients with sequelae of stroke who met the inclusion criteria were included. They received conventional treatment and in addition also received treatment with the MEXO monoarticular robot three times a week during 6 weeks. Each session consisted of 10 min of passive stretching followed by 20 min of active movement training with visual feedback (10 min active without resistance, 10 min with resistance) and a final phase with 10 min of passive stretching. The following variables were measured pre– and post–treatment: joint range of motion and ankle muscle strength, monopodal balance, muscle tone, gait ability and satisfaction with the use of assistive technology.

**Results:** Statistically significant improvements were obtained in joint range measured by goniometry and in balance measured by monopodal balance test. Also in walking capacity, through the measurement of travelled distance.

**Discussion and significance:** Device usability and patient safety were tested. Patients improved joint range and monopodal balance. The MEXO exoskeleton might be a good alternative for the treatment of spastic ankle joint in people with a stroke sequela.

## 1 Introduction

Stroke is a global health problem due to its high mortality rate and level of physical and mental disability. According to the World Health Organization, stroke is the third leading cause of death in first world countries, after heart disease and cancer. It is also the second leading cause of death in geriatric patients and is the leading cause of disability worldwide. Every year, 15 million individuals suffer a stroke, of whom 5.5 million die and another 5 million are left with a lifelong disability. In 2015, an estimated 17.7 million people died from cardiovascular causes, accounting for 31% of all deaths worldwide. Of these deaths, 7.4 million were due to coronary heart disease, and 6.7 million were due to cardiovascular diseases (World Health Organization, 2017).

Stroke is defined as “an episode of acute neurological dysfunction presumed to be caused by ischemia or haemorrhage, persisting ≥ 24 h or until death” (Sacco et al., 2013). Once a stroke has occurred, hemiparesis and spasticity are usually the most common sequela [there is a big panoply of secondary complications though (Teasell, 1992)]. Focal neurological deficits in the acute phase compromise the mobility of patients, with a notable reduction in physical activity and, therefore, in physical condition. Immobility and inactivity cause loss of muscle mass, increased body fat, limited joint mobility and reduced bone mineral density. Ankle spasticity is one of the most common movement disorders after stroke. Spastic hypertonic ankles can severely decrease patients’ mobility and independence (Zhou et al., 2015).

The basic mechanism of muscle tone control is the myotatic reflex which is originated by activation of the primary afferent endings of the neuromuscular spindle and mediated by the spinal cord (Liddell and Sherrington, 1924). The discharge of the primary endings has both static and dynamic components that give rise to the tonic and phasic components of the stretch reflex. Spasticity develops when, due to injury, the stretch reflex arc is isolated from its supraspinal modulatory system resulting in abnormal excitation of alpha and gamma motor neurons (Spaich and Tabernig, 2002).

Physical therapy for the rehabilitation of patients with spastic ankle consists of providing repetitive exercises for the ankle muscles, so that plantar flexion and dorsiflexion lead to the reintegration of the distance between the feet during the swing phase of the gait cycle (Smania et al., 2010). However, the ankle is an anatomical complex that produces joint movements with two degrees of freedom: dorsi/plantarflexion and inversion/eversion (Jones, 2019). Repetitive therapy sessions are required to restore lost motion in terms of joint ranges and forces (Hussain et al., 2017).

Conventional physiotherapy has evolved from techniques focused on strengthening and analytical movement practice, to approaches focused on regaining functional movements, such as gait through training, and the use of, for example, treadmills with partial body weight support (Belda-Lois et al., 2011). Robots have been developed and have begun to be applied in the field of biomedicine. These robotic–assisted devices used in gait rehabilitation in patients with neurological pathologies have achieved good results in the recovery of lower limb functionality, they seem to have decreased the physical effort made by therapists and intensified gait training in patients Calderón-Bernal et al. (2015). According to the type of structure, the authors define a monoarticular robotic therapeutic “exoskeleton” as an electromechanical device which closely fits the user’s body and is designed to mobilize one human joint through a physiological range of motion to train the recovery of user’s capacity.

This type of robotic exoskeletons that focus on a single joint can therefore facilitate movements and also help to correct vicious postures. As one of the most common post–stroke sequelae is increased tone and consequent decreased mobility at the ankle joint, there are exoskeletons which are designed to correct the equinus position of the foot and assist its movement, such as the ankle robot or anklebot (Calderón-Bernal et al., 2015; Forrester et al., 2011), which position the foot during the swing phase and assist in facilitating foot mobility, and can be used in standing, seated and supine positions, as well as an interactive video game system where the foot movement is visualised (Chang et al., 2017).

Another prototype used in ankle rehabilitation is MAFO, a motorised ankle and foot orthosis, capable of performing dorsiflexion and plantar flexion movements and providing visual biofeedback on electromyographic signals; this device has been designed as a tool for recovery, simplification and improvement of motor learning thus facilitating ankle functionality, and not developed to be a walking orthosis (Asín-Prieto et al., 2013).

In gait rehabilitation, it is worth mentioning the importance of motor control of the ankle, as several studies applying robotic therapy have shown improvements in the performance of the joint, pointing out by the authors that the motor ability of the ankle could be improved, explained by the increase in motor cortical excitability for the tibialis anterior, thus achieving voluntary control of the ankle flexor musculature and an increase in the flexo–extension of the ankle (Calderón-Bernal et al., 2015).

Recently, significant research efforts have been made to improve the mechanism design, actuation, control algorithms and interaction for these robotic orthoses and parallel ankle robots (Jimenez-Fabian and Verlinden, 2012; Kwon et al., 2019; Asín-Prieto et al., 2020). The goal behind the research is that the design of these robotic devices should provide natural movement patterns for patients with neurological disorders, and the actuation system used with these robots should provide safe and efficient human–robot interaction. The goal of developing advanced control algorithms is to personalise robotic assistance according to the level of disability and stage of rehabilitation of the patient (Hussain et al., 2017).

Miao et al. (2018) reviewed the designs of existing ankle rehabilitation robots. They reported that while most robotic–assisted ankle rehabilitation techniques have been shown to be effective for ankle physiotherapy, they might have design drawbacks that have prevented their wide–range applications. Their review states that an optimal ankle rehabilitation robot design should be characterised with a centre of rotation aligned with the ankle joint, that the number of ranges of motion depends on specific applications, and that the single range of motion robot is developed especially for ankle stretching along dorsi– and plantarflexion; while multiple ranges of motion devices are more suitable for complete ankle rehabilitation exercises. They also add that the adjustability of the system, its method of fixation to the users’ foot, and the mechanical stops, affect their clinical application.

In a comprehensive review of recent developments in the field of robotic–assisted ankle rehabilitation, significant advances in mechanism design, control, and experimental evaluations of ankle rehabilitation robots are reported. However, the authors consider that it is necessary to develop improved methods for motion detection and definition of reference trajectories, as well as more objective quantitative evaluations that must be performed to establish the clinical importance of these robots (Hussain et al., 2021).

The device used in this study is the Motorised ankle-foot EXOskeleton (MEXO), a research prototype device developed at the Neural Rehabilitation Group at Cajal Institute of the Spanish National Research Council (CSIC) in Spain.

Given the high prevalence of patients with post–stroke hemiplegia attending the Rehabilitation Service of the J. N. Lencinas Hospital (Mendoza, Argentina), it is of interest to study the effects of possible treatments to improve the functionality of the affected ankle, thus improving walking in them. So far, the most commonly used treatment in this service to improve forefoot drop in hemiplegic patients has been conventional physio–kinesiotherapy, ankle–foot orthosis and functional electrostimulation (FES).

The development of this work was part of the REASISTE Research Network (Ibero–American Network for rehabilitation and assistance of patients with neurological damage using low–cost robotic exoskeletons), funded by CYTED (Ibero–American Programme of Science and Technology for Development) and aimed to contribute to the validation and safety of cost-effective robotic rehabilitation equipment and its possible transfer to treatment spaces.

## 2 Objective

The main objective of the study is to measure the effects of passive stretching, combined with active and resisted movement, on the ankle and its impact on gait functionality, accompanied by visual feedback, by means of an interactive playful software using low–cost Monoarticular robotic EXOskeleton MEXO in patients with sequelae of stroke and spastic ankle; in the Rehabilitation Service of the J. N. Lencinas Hospital (Mendoza, Argentina) during the period 2020–2021. Secondary objectives are to evaluate pre– and post–treatment ankle variables such as joint range, muscle strength, muscle tone, functional gait performance (speed–distance–fall risk–walking ability), to check the usability of the device and patient safety, and also to measure the degree of patient satisfaction with this assistive technology.

## 3 Materials and methods

This is an open, pre–post, uncontrolled, non–randomised, 6–week, quasi–experimental study. The study population is made up of patients attending the Rehabilitation Service who present with hemiparesis following a stroke, with an evolution time of more than 3 months, during the period 2020–2021.

### 3.1 Protocol

All included patients received conventional treatment and treatment with the MEXO exoskeleton three times a week for 6 weeks. The conventional rehabilitation treatment, lasting 60 min, consisted of passive mobilizations, active exercises and strength training with isotonic exercises. Also, proprioceptive training and balance exercises. Following this treatment, they received treatment with MEXO with each session consisting of 10 min of passive stretching followed by 20 min of active movement training with playful visual feedback (10 min active without resistance, 10 min with resistance) and a final 10-min phase of passive stretching.

Pre–treatment, during treatment sessions, and post-treatment data were collected for the variables listed in Table 1.

**TABLE 1:** Variable description.

Variable	Concept	Measuring instrument
Ankle joint range	The angle in degrees from the start point to the end point of the movement	Goniometer
Ankle muscle strength	Ability of a muscle to exert tension against a load during muscle contraction	Medical Research Council (MRC) Score 0 to 5
Monopodal balance	The time in seconds that the patient can stand on one foot or the other	Time in seconds
Muscle tone	Permanent state of partial, passive and continuous contraction of muscles	Modified Ashworth Scale. Score from 1 to 5
Satisfaction with the use of Assistive Technology	Satisfaction in relation to health can be defined as an attitude about a service, product, a service provision or an individual’s state of health, according to expectations, wishes or needs.	QUEST scale. Items 1 to 5.
Walkability:		
- Distance travelled	Distance in metres travelled in a set period of time	2 min test
- Time	Time it takes to cover a given distance	10-m test
- Walking ability	Assessment of a subject’s chances of successfully completing an ambulation	Timed up and go; and time and items from 1 to 5

### 3.2 Ethical considerations

The study was evaluated and approved by the Teaching and Research Committee of the José Nestor Lencinas Hospital and by the COPEIS (Provincial Health Research Ethics Committee) of the province of Mendoza (Argentina).

All the patients were informed about their participation in the study. All stroke survivors in the Rehabilitation Service who met the inclusion criteria and who did not meet the exclusion criteria were eligible to participate in this study. All patients who participated in the study agreed to the treatment with the MEXO exoskeleton.

The following inclusion criteria was considered: hemiparesis resulting from a single stroke event, age between 18 and 65 years old, post–stroke time equal or greater than 3 months, independent walking, passive ankle joint range at dorsiflexion of at least 90 degrees, dorsiflexor muscle strength of at least two according to Medical Research Council (MRC) index, ankle plantarflexor spasticity of ± 2 according to Modified Ashworth Scale, ability to understand and follow instructions, informed consent form signed. The exclusion criteria includes comorbidities affecting gait, history of frequent falls, debilitating or immunosuppressive disease, alteration of mental functions that prevent the patient from following instructions.

### 3.3 Description of the equipment

The MEXO device has been developed at the Neural Rehabilitation Group of Cajal Institute CSIC in the scope of the REASISTE Iberoamerican Network that supports the development of cost-effective robotic solutions for therapy. It consists of a mobile platform adjustable to different anthropometries that allows dorsal and plantar flexion of the right or left foot, by means of a manual clutch in the motor–gearbox assembly. It has a position sensor of the ankle angle and measures the force that the motor exerts. It also has adjustable mechanical stops with screws that prevent the platform from reaching extreme positions, which are previously defined by the therapist before starting the treatment by means of passive mobilisation. As a safety measure, the device is equipped with an emergency stop switch, which interrupts the power supply to the entire device.

MEXO is intended for controlling the movement of the human ankle providing an improved visual feedback by means of interactive software (video game) aimed at people with neuromotor impairments such as cerebral palsy, stroke and incomplete spinal cord injury. The exoskeleton is a stationary system that allows active/passive mobilisation of the ankle in the sagittal plane, allowing the required stretching exercises to be performed in such a way that the angular trajectory of the user’s ankle follows a specific trajectory depending on the video game with which it is synchronised. The MEXO device is used in a seated position, and has ergonomic adaptations for contact, support, alignment and fixation to each user’s limb.

**FIGURE 1:**
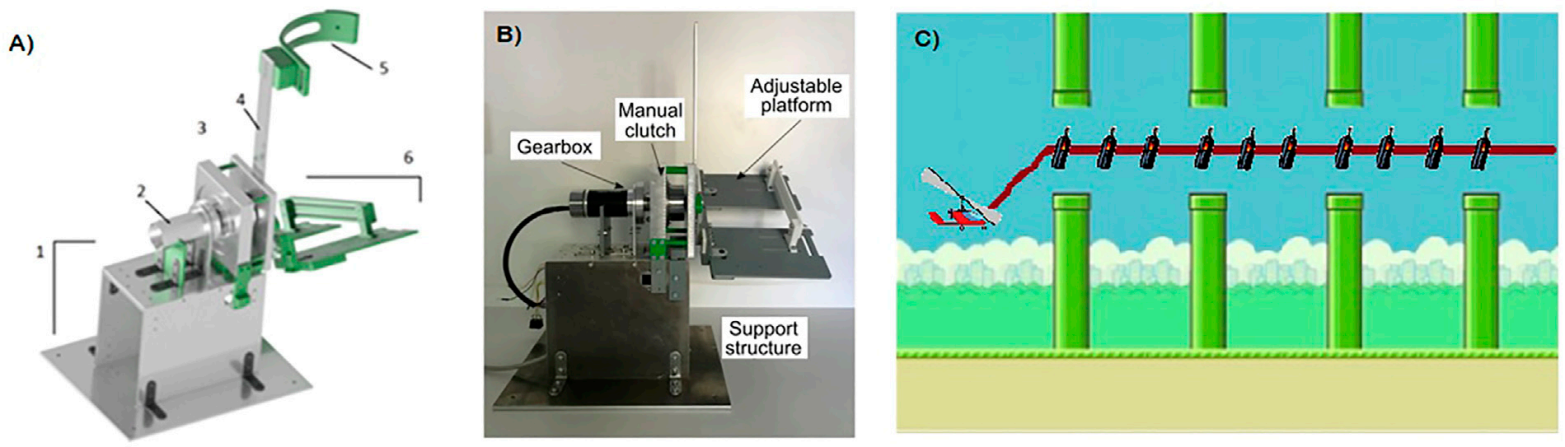
Structural components and visual paradigm of MEXO: **(A)** Mechanical components, 1- structural support, 2- motor and gearbox assembly, 3- clutch, 4- leg structure bar, 5- strap and 6- insole; **(B)** Front view of the MEXO robot; and **(C)** Visual interface.

The exoskeleton has three operation modes:

• Passive mode: The user’s ankle follows the movement rigidly imposed by the exoskeleton, thus leading to passive stretching. In this mode, the actuator clutch is engaged. Thus, the system imposes a joint trajectory within the limits of the subject’s joint assessed in the passive joint evaluation. The maximum range of motion in this mode goes from a dorsiflexion of 25 degrees up to a plantarflexion of 55 degrees, with a total of 80 degrees travel, and it is possible to adjust extreme limits in case of joint limitation by the patient, for which the robot’s travel must correspond to the passive joint evaluation. The speed of the movement goes from a maximum full range travel time of 6 s (slow speed, 13.33 degrees/second) taking into account the speed–spasticity ratio, to a minimum of 2 s (fast speed, 40 degrees/second).
• Active mode: The exoskeleton actuator would not exert any force, and the user has to actively perform the stretches. In this mode, the actuator clutch is not engaged, and the work is performed mainly on the flexor muscles of the foot (tibialis anterior and peroneus) due to the effect of gravity, and secondarily on the extensors (calf and soleus) in the search for maximum joint amplitude. The range of motion in this mode also goes from a dorsiflexion of 25 degrees up to a plantarflexion of 55 degrees, with a total of 80 degrees travel, and it is possible too to adjust extreme limits in case of joint limitation by the patient, for which the robot’s travel must correspond to the active muscle evaluation. The speed of the movement can be regulated (within the limits set for the passive mode) according to the patient’s muscular resistance. In the visual paradigm bio-feedback, the gyrocopter (Figures 1C) goes up and down with the dorsi/plantarflexion, and the efficiency or error in the movement produced by the patient is recorded. The challenge of this mode is to achieve good score, and sustain this correct and wide movement over time (overcoming the possible fatigue).
• Resistive mode: In this case, the exoskeleton actuator introduces disturbances to the movement of the visual trajectory, so that the user must perform movements that counteract these disturbances in order to try to complete the trajectory as accurately as possible. In this mode, the actuator clutch is engaged. The system applies a single resistance pattern to the movement that simulates the sagittal force profile of ground reaction during the stance phase of the gait, and performs a pause (rest) that would correspond to the swing phase. The work is mainly on the extensor muscles (calf and soleus) by making an eccentric–concentric contraction simulating muscle activation during the stance phase of gait. The challenge of this mode is to achieve a good score and sustain this movement pattern over time against the perturbations. The range of motion in this mode goes from a dorsiflexion of 15 degrees (the robot overcomes the patient’s strength –eccentric–) up to a plantarflexion of 25 degrees (patient overcomes robot force –concentric–). With a total of 40 degrees travel, and a unique movement pattern that simulates the stance phase of gait and a pause (rest) that would correspond to the swing phase, it is possible to adjust extreme limits in case of joint limitation by the patient, for which the robot’s range of movement must correspond to the active muscle evaluation. The speed of the movement can be regulated (within the limits set for the passive mode) according to the patient’s muscular resistance, and in this “Resistive mode”, the resistance offered by the robot to the patient can also be regulated (from five to 20 N); these parameters are adjusted according to the patient’s muscular resistance. As in the previous mode, the visual paradigm bio-feedback shows the gyrocopter going up and down with dorsi/plantarflexion, and the efficiency or error in the movement produced by the patient against the resistance given by the robot is recorded, with pause cycles shown on the screen with the word “Descansa” (Rest in Spanish).

The main components of the exoskeleton (depicted in Figures 1A,B) are the following:

• Structural support: The structure consists of a series of aluminium plates that form the main support for the exoskeleton and raise it above the ground surface. The base of this support can be anchored to the ground by means of screws fixed or a counterweight fixed to the base.
• Motor and gearbox assembly: The exoskeleton actuator consists of a brushless direct current motor, coupled to a planetary gearbox (Maxon Motor, www.maxongroup.com). Both components were specifically selected so that the actuator can develop the required torque on the patient’s ankle during rehabilitation exercises.
• Clutch: The actuator has a clutch with a manual disengagement/engagement system attached to the motor and side panels. When the system is disengaged, the ankle joint of the exoskeleton moves freely with the user’s foot, while when the system is engaged it is the motor–gearbox assembly that executes this movement.
• Leg structure bar: On this bar the height position of the strap is adjusted so that the adaptation of the exoskeleton on the user’s leg is optimal.
• Strap: Component that embraces the user’s leg and is adjusted to it by means of a BOA fastener, so that the exoskeleton is adapted to the user’s leg.
• Insole: This component, made up of two platforms, is adjustable in width and length so that it can be adapted to different foot sizes depending on the user who is going to use the device. The foot, with the user’s footwear is attached to the insole by means of several Velcro straps and BOA fasteners.

The equipment includes safety measures both at an electronic level (emergency stop switch, which interrupts the power supply to the entire device) and at a mechanical level (physical stops limiting ankle dorsi–plantarflexion).

MEXO is connected to a standard PC which contains the set of software functions to perform the therapy. The computer receives information from the MEXO’s position sensor to move the game avatar through the virtual scenario. The avatar’s range of motion is adapted according to the patient’s joint range capabilities, so as not to overly penalise its performance. In addition to the avatar’s position, the game sets the motor resistance when the “resisted” game mode is selected. The software that integrates the device is divided into two fundamental elements: control and visual paradigm. The control is executed on the control board of the device (Arduino MEGA), and is responsible for: 1) reading the current on the motor and the angle, 2) sending this information *via* serial port to the computer that executes the visual interface (implemented in MATLAB), and 3) controlling the motor for the different operating modes. The visual paradigm, shown in Figure 1C, is implemented in such a way that it receives the angle information from the control board of the device, and moves the item on the screen according to this information (dorsiflexion and plantarflexion move the gyrocopter up and down respectively). It is also responsible for sending the corresponding commands for the different control modes.

### 3.4 Participants

Patient demographics are shown in Table 2. 16 patients were evaluated, with finally nine completing the entire treatment. Five patients had to drop out due to restrictions imposed by the Ministry of Health, Social Development and Sports due to the COVID–19 pandemic situation and two for complications with transportation in the area. The mean age of the patients was 54.9 years ranging from 42 to 64 years; four were female and five male; the mean time since stroke was 7.1 months ranging from three to 20 months. Only two patients required walking assistance. There were three patients with left, and six with right, hemibody involvement, four with haemorrhagic and five with ischaemic stroke.

**TABLE 2:** Clinical and demographic characteristics of patients.

	P1	P2	P3	P4	P5	P6	P7	P8	P9
Age	58	57	54	53	42	60	50	56	64
Gender	F	M	M	F	F	M	M	M	F
Stroke evolution time in months	8	6	7	3	3	3	20	10	4
Walking assistance	No	No	No	No	No	No	Cane	Cane	No
Affected hemibody	Left	Right	Right	Left	Right	Right	Right	Right	Left
Type of stroke	H	I	I	I	I	I	H	H	H
Spasticity in flexion	0	1	1	0	0	0	1	0	0
Spasticity in extension	0	1	1	0	0	0	0	0	0

### 3.5 Data analysis

With the data obtained, a descriptive and hypothesis testing analysis was carried out comparing the changes in the variables collected before and after the treatment (pre–post analysis) using the Wilcoxon test. We also conducted correlation analyses (Pearson and Spearman, depending on normality tests –Kolmogorov-Smirnov-based Lilliefors test–, i.e., Pearson for normally distributed variables on both components of the pair, Spearman otherwise).

## 4 Results

The results of the variables studied pre– and post–treatment are summarised in Table 3.

**TABLE 3:** Variables studied pre and post treatment.

Patient	Goniometry (deg)				Spast. (Ash)		Muscle strength (MRC)		Monopodal balance test (deg)		T2min (m)	T10m (sec)	TUGT (sec)	PROM (deg)	AROM (deg)	AScore (%)	ARMSE (deg)	RScore (%)	RRMSE (deg)
	Flex (+)		Ext (−)		F	E	F	E	Right	Left									
	FP	A	FP	A															
1-PRE	5	2	54	50	0	0	−4/5	−4/5	40	19	157	6	7	67.16	72.98	74.59	5.72	67.86	6.80
1-POST	17	13	48	60	0	0	+4/5	+4/5	59	23.49	165	7.29	7	80.69	75.23	86.51	5.18	76.75	6.51
2-PRE	0	0	40	38	1	1	−4/5	−4/5	10	38	160	8	6	75.65	62.64	85.56	5.05	75.29	7.43
2-POST	0	3	41	41	1	1	4/5	4/5	12	34	160	6	6	81.19	64.93	91.72	3.18	83.69	4.05
3-PRE	5	5	38	38	1	1	−4/5	−4/5	6	5	157	6	6	63.51	64.33	82.59	4.58	76.04	5.43
3-POST	10	10	50	40	0	0	+4/5	+4/5	40	30	188	5.32	6	77.93	73.50	95.57	3.29	98.80	2.65
4-PRE	3	2	45	50	0	0	3/5	3/5	6	2	157	9.39	10.32	71.81	71.37	80.19	5.37	68.91	6.26
4-POST	25	20	60	60	0	0	+4/5	+4/5	40	17.5	160	5.65	6.56	78.20	83.04	99.80	3.02	97.81	3.70
5-PRE	0	0	30	25	0	0	+4/5	+4/5	5.6	4.11	160	8.68	6.9	73.97	74.05	87.06	4.53	83.58	4.87
5-POST	15	15	35	35	0	0	+4/5	+4/5	12.58	16.8	180	5.84	2.74	83.60	71.71	101.49	2.55	94.68	3.70
6-PRE	10	0	65	60	0	0	−4/5	+4/5	1.3	2.9	89	9.6	10.7	69.92	9.19	66.01	7.48	55.46	9.25
6-POST	10	0	65	60	0	0	+4/5	+4/5	4.20	8.69	111	8.6	9.8	65.62	174.66	100.72	1.92	83.33	3.92
7-PRE	10	10	35	35	1	0	4/5	4/5	1.99	18.05	107	9.14	12.7	61.18	44.95	55.16	6.50	51.62	5.90
7-POST	0	5	50	50	1	0	+4/5	+4/5	3.5	30.5	123	8.3	10.58	57.27	50.03	79.05	4.80	54.36	7.54
8-PRE	3	3	40	40	0	0	4/5	4/5	1.03	1.09	100	7.6	8.99	64.59	58.26	59.74	7.93	62.54	5.89
8-POST	14	10	50	45	0	0	+4/5	+4/5	1	1.53	130	6.54	9.02	73.58	53.59	84.39	6.93	87.35	4.65
9-PRE	7	10	30	50	0	0	+4/5	4/5	1	-	70	12.6	12.78	76.43	72.44	46.67	9.49	38.28	13.82
9-POST	15	10	42	47	0	0	5/5	5/5	0.91	0.72	82	13.6	14.1	66.92	39.80	65.58	5.94	49.18	10.80

FP, forced passive; A, active; T2min, 2 min walking test; T10m, 10 m walking test; TUGT, timed up and go test; deg, degrees; sec, seconds; m, metres; PROM, passive range of motion; AROM, active range of motion; AScore, active score; ARMSE, active root mean squared error; RScore, resistive score; RRMSE, resistive root mean squared error; Spast., spasticity; Ash, ashworth; F, flex; E, ext.

Pre– and post–intervention comparisons were made in the patient group for all variables. The mean, standard deviation, median, minimum, maximum, mode and normality values are expressed in Table 4; and the pre– and post–treatment statistical significance in Table 5.

**TABLE 4:** Values of mean (AVG), standard deviation (STD), median (MDN), minimum (MIN), maximum (MAX), mode (MOD) and normality (NRM) pre- and post-treatment of the variables.

		GFFP	GFA	GEFP	GEA	AshwF	AshwE	MSF	MSE	RMBT	LMBT	T2min	T10m	TUGT	PROM	AROM	AScr	ARMSE	RScr	RRMSE
AVG	PRE	4.22	3.56	41.89	42.89	0.33	0.22	0.33	1.22	8.10	10.52	128.56	8.56	9.04	69.36	58.91	70.84	6.30	64.40	7.29
	POST	11.78	9.56	49.00	48.67	0.22	0.11	4.11	4.11	19.24	18.14	144.33	7.46	8.31	73.89	76.28	89.42	4.09	80.66	5.28
STD	PRE	3.19	4.00	11.42	10.51	0.50	0.44	4.12	3.93	12.35	12.30	36.53	2.02	2.72	5.54	20.85	14.56	1.70	14.07	2.76
	POST	8.00	6.19	9.29	9.51	0.44	0.33	0.33	0.33	21.45	12.47	34.92	2.58	2.82	8.82	39.40	11.91	1.69	17.95	2.57
MDN	PRE	5.00	2.00	40.00	40.00	0.00	0.00	3.00	4.00	5.60	4.56	157.00	8.68	8.99	69.92	64.33	74.59	5.72	67.86	6.26
	POST	14.00	10.00	50.00	47.00	0.00	0.00	4.00	4.00	12.00	17.50	160.00	6.54	7.00	77.93	71.71	91.72	3.29	83.69	4.05
MIN	PRE	0.00	0.00	30.00	25.00	0.00	0.00	−4.00	−4.00	1.00	1.09	70.00	6.00	6.00	61.18	9.19	46.67	4.53	38.28	4.87
	POST	0.00	0.00	35.00	35.00	0.00	0.00	4.00	4.00	0.91	0.72	82.00	5.32	5.74	57.27	39.80	65.58	1.92	49.18	2.65
MAX	PRE	10.00	10.00	65.00	60.00	1.00	1.00	4.00	4.00	40.00	38.00	160.00	12.60	12.78	76.43	74.05	87.06	9.49	83.58	13.82
	POST	25.00	20.00	65.00	60.00	1.00	1.00	5.00	5.00	59.00	34.00	188.00	13.60	14.10	83.60	174.66	101.49	6.93	98.80	10.80
MOD	PRE	5.00	0.00	30.00	50.00	0.00	0.00	−4.00	4.00	6.00	1.09	157.00	6.00	6.00	61.18	9.19	46.67	4.53	38.28	4.87
	POST	0.00	10.00	50.00	60.00	0.00	0.00	4.00	4.00	40.00	0.72	160.00	5.32	6.00	57.27	39.80	65.58	1.92	49.18	2.65
NRM	PRE	Yes	Yes	Yes	Yes	No	No	No	No	No	No	No	Yes	Yes	Yes	Yes	Yes	Yes	Yes	Yes
	POST	Yes	Yes	Yes	Yes	No	No	No	No	No	Yes	Yes	Yes	Yes	Yes	No	Yes	Yes	Yes	Yes

GFFP, goniometry flexion FP; GFA, goniometry flexion A; GEFP, goniometry extension FP; GEA, goniometry extension A; AshwF, ashworth flexion; AshwE, ashworth extension; MSF, muscle strength-flexion; MSE, muscle strength extension; RMBT, right monopodal balance test; LMBT, left monopodal balance test; T2min, 2 min walking test; T10m, 10 m walking test; TUGT, timed up and go test; PROM, passive ROM; AROM, active ROM; ASCr, active score; ARMSE, active RMSE; RScr, resistive score; RRMSE, resistive RMSE.

**TABLE 5:** *p*-value of treatment variables (*p < 0.05).

Variables	Signedrank	*p* value
Goniometry flexion FP	4.00	*p* = 0.0469*
Goniometry flexion A	2.50	*p* = 0.0625
Goniometry extension FP	3.00	*p* = 0.0391*
Goniometry extension A	2.50	*p* = 0.0312*
Ashworth flexion	1.00	*p* = 1
Ashworth extension	1.00	*p* = 1
Muscle strength flexion	0.00	*p* = 0.0312*
Muscle strength extension	0.00	*p* = 0.0625
Right monopodal balance test	3.00	*p* = 0.0195*
Left monopodal balance test	5.00	*p* = 0.0391*
2 min walking test	0.00	*p* = 0.0078*
10-m walking test	35.50	*p* = 0.1367
Timed up and go test	16.00	*p* = 0.3125
Passive range of motion	9.00	*p* = 0.1289
Active range of motion	15.00	*p* = 0.4258
Active score	0.00	*p* = 0.0039*
Active RMSE	45.00	*p* = 0.0039*
Resistive score	0.00	*p* = 0.0039*
Resistive RMSE	41.00	*p* = 0.0273*

**FIGURE 2:**
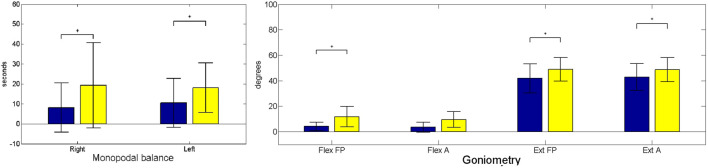
Monopodal balance (left subfigure) and goniometry (right subfigure) mean values. Where A, Active; FP, Forced Passive; Ext, Extension; and Flex, Flexion. Statistically significant changes are denoted with an asterisk (*). Blue (black if printed in greyscale) corresponds to preintervention; yellow (light grey in greyscale) corresponds to postintervention moment.

**FIGURE 3:**
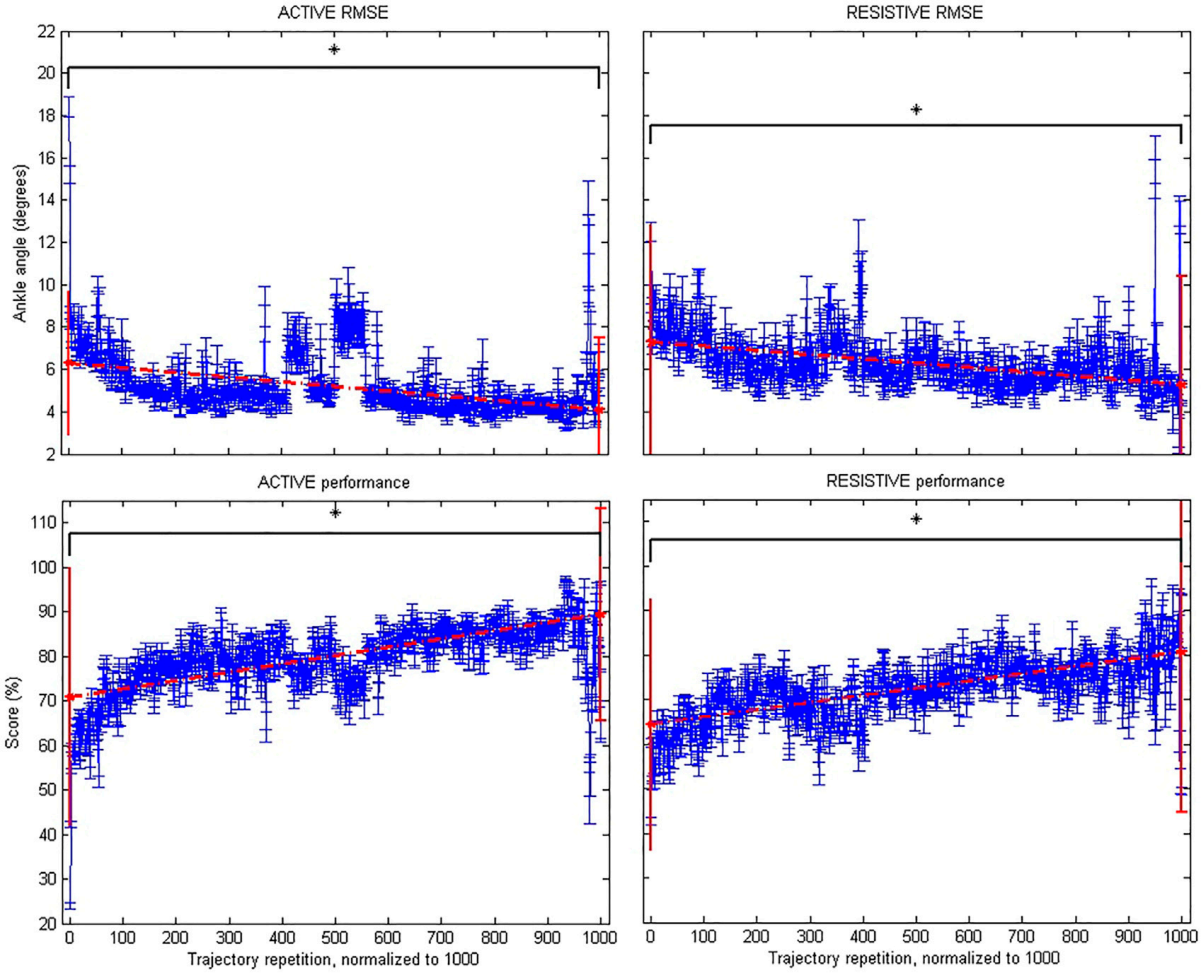
Robotic metrics: Active root mean squared error (upper left subfigure); Active performance (lower left); Resistive root mean squared error (upper right); and Resistive performance (lower right). Statistically significant changes are denoted with an asterisk (*).

Pre–post intervention comparisons in the patient group were plotted for the variables “goniometry”, “monopodal balance”, “active root mean squared error”, “active performance”, “resistive root mean squared error” and “resistive performance”. Comparisons of the intervention effect in the group (variables collected pre– and post–treatment pre–post analysis using the Wilcoxon test) for these two variables are presented in Figure 2 for “monopodal balance” and “goniometry”. Mean values with standard deviation, together with mean and deviation for the values corresponding to the first and last score and rmse value (normalized to 1,000 repetitions) for the intrasubject linear fitting for the variables “active root mean squared error”, “active performance”, “resistive root mean squared error” and “resistive performance” are presented in Figure 3. We used the measurements before and after the training days to perform the statistical analyses.

Table 5 shows the pre– and post–intervention statistical inference. The results of the study showed significant changes in monopodal balance, as well as in FP flexion, FP extension, A extension kinematics, together with the 2-min walking test, and muscle strength during flexion. Furthermore, we also found significant changes in score and root mean squared error for both active and resistive training paradigms.

We also evaluated the User Satisfaction with Assistive Devices Technology by means of the QUEST Quebec Survey version 2.0 (Demers et al., 2002) adapted to the MEXO exoskeleton. Two unrelated questions were removed from the “Device” section, and another two from the “Services” section. Unrelated dimensions that were removed with respect to the apparatus are dimensions and weight. Regarding the service, the delivery process dimension and repair and maintenance were also eliminated (refer to Table 6 for results).

**TABLE 6:** Results of the QUEST questionnaire.

Assessed dimensions	Patient #	P1	P2	P3	P4	P5	P6	P7	P8	P9
“Device” section	Ease of adjustment	4	4	5	5	5	4	5	4	4
	Safety	5	5	5	5	5	4	5	5	3
	Durability	5	4	4	5	5	5	5	5	4
	Ease of use	5	5	5	5	5	5	5	5	5
	Comfort	5	5	5	5	5	5	5	5	5
	Effectiveness	5	5	5	5	5	5	5	5	3
“Services” section	Quality	5	5	5	5	5	5	5	5	5
	Follow–up	5	5	5	5	5	5	5	5	5
“Additional questions”	Overall satisfaction level with the device	5	4	4	5	5	4	5	5	4
	Overall satisfaction with services	5	5	5	5	5	5	5	5	5
“Selection of three most important questions”		- S	- F	- EU	- EU	- EU	- S	- S	- P	- C
		- Co	- EU	- S	- Co	- Co	- Co	- Ef	- EU	- EU
		- P	- Ef	- Ef	- Ef	- Ef	- Ef	- EU	- Co	- Cs

S, safety; Co, comfort; P, professional assistance; F, fit; EU, ease of use; Ef, effectiveness; Cs, continuous service.

**FIGURE 4:**
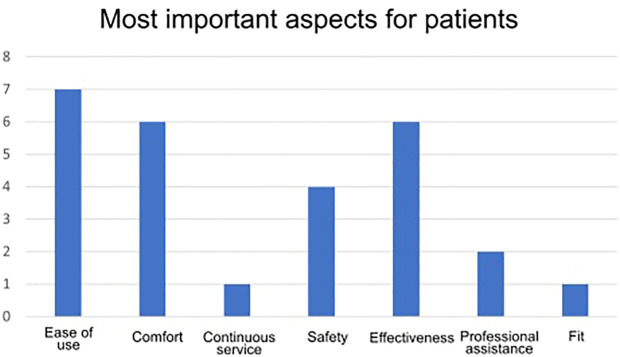
Most important aspects for patients.

Figure 4 shows the most important aspects for the patients, extracted from the questionnaires.

Table 7 shows the mean, standard deviation, median, minimum, maximum, mode and normality values for QUEST results.

**TABLE 7:** Values of mean, standard deviation, median, minimum, maximum, mode and normality of QUEST results.

EA	S	D	EU	Co	Ef	Q	Fu	OD	OS
Average									
4.44	4.67	4.67	5.00	5.00	4.78	5.00	5.00	4.56	5.00
Standard deviation									
0.53	0.71	0.50	0.00	0.00	0.67	0.00	0.00	0.53	0.00
Median									
4.00	5.00	5.00	5.00	5.00	5.00	5.00	5.00	5.00	5.00
Minimum									
4.00	3.00	4.00	5.00	5.00	3.00	5.00	5.00	4.00	5.00
Maximum									
5.00	5.00	5.00	5.00	5.00	5.00	5.00	5.00	5.00	5.00
Mode									
4.00	5.00	5.00	5.00	5.00	5.00	5.00	5.00	5.00	5.00
Normality									
No	No	No	No	No	No	No	No	No	No

EA, ease of adjustment; S, safety; D, durability; EU, ease of use; Co, comfort; Ef, effectiveness; Q, quality; Fu, follow-up; OD, overall satisfaction level with the device; OS, overall satisfaction level with services.

Tables 8, 9 show the correlations with a value of p greater than 0.05 (*p* > 0.05) for both Spearman and Pearson correlation tests respectively. Spearman has been applied to those data that do not comply with normality (both variables, or at least one of them). Pearson has been applied to those variable pairs where both comply with normality. Correlations for EU (Ease of Use), Co (Comfort), Q (Quality), Fu (Follow-up) and OS (Overall satisfaction with Services) could not be performed, due to they having null variance (all patients scored 5 points on all these variables). We observed moderate to strong correlations in all the correlating pairs. Some interesting correlations are those that find increased monopodal balance for increased active ROM; decreased Timed Up and Go Test time for increased root mean squared error in the resistive paradigm; or decreased score for the 10 m walking test for both active and resistive scores.

**TABLE 8:** Spearman correlations table (*p* > 0.05) for PRE and POST–treatment variables.

Variable 1	Variable 2	Spearman correlation coefficient
AROM POST	AScore POST	−0.73
T10m POST	S	−0.86
T2min POST	S	−0.91
RMBT POST	AROM POST	0.88
RMBT POST	T2min POST	−0.75
T10m POST	S	−0.73
T2min POST	S	0.73
RMBT POST	AROM POST	0.70
RMBT POST	T2min POST	0.81
RRMSE PRE	S	−0.73
RScore PRE	RMBT POST	0.69
ARMSE PRE	RMBT POST	−0.72
T10m PRE	S	−0.73
T2min PRE	S	0.75
T2min PRE	PROM POST	0.86
T2min PRE	TUGT POST	−0.91
T2min PRE	T10m POST	−0.74
T2min PRE	T2min POST	0.82
T2min PRE	RScore PRE	0.89
T2min PRE	ARMSE PRE	−0.92
T2min PRE	AScore PRE	0.91
T2min PRE	TUGT PRE	−0.82
LMBT PRE	LMBT POST	0.73
RMBT PRE	T2min POST	0.73
RMBT PRE	LMBT POST	0.75
RMBT PRE	RMBT POST	0.88
RMBT PRE	ARMSE PRE	−0.70
RMBT PRE	TUGT PRE	−0.69
RMBT PRE	T2min PRE	0.79
MSE PRE	RMBT POST	−0.75
MSE PRE	RMBT PRE	−0.88
MSF PRE	MSE PRE	0.77
AshwF PRE	LMBT POST	0.82
AshwF PRE	GFFP POST	−0.79
GEFP PRE	AROM POST	0.76
GFFP PRE	T2min PRE	−0.70

AROM, active range of motion; AScore, active score; T10m, 10 m walking test; S, safety; T2min, 2 min walking test; RMBT, right monopodal balance test; RRMSE, resistive root mean squared error; RScore, resistive score; ARMSE, active root mean squared error; PROM, passive range of motion; TUGT, timed up and go test; LMBT, left monopodal balance test; MSE, muscle strength extension; MSF, muscle strength-flexion; AshwF, ashworth flexion; GFFP, goniometry flexion FP.

**TABLE 9:** Pearson correlations table (*p* > 0.05) for PRE and POST–treatment variables.

Variable 1	Variable 2	Pearson correlation coefficient	Variable 1	Variable 2	Pearson correlation coefficient
RRMSE POST	RScore POST	−0.94	RScore POST	AScore PRE	0.82
RRMSE POST	AScore POST	−0.92	AScore POST	AScore PRE	0.81
RScore POST	AScore POST	0.88	PROM POST	AScore PRE	0.83
ARMSE POST	AScore POST	−0.81	TUGT POST	AScore PRE	−0.96
RScore POST	PROM POST	0.72	T10m POST	AScore PRE	−0.82
RRMSE POST	TUGT POST	0.84	T2min POST	AScore PRE	0.91
RScore POST	TUGT POST	−0.85	RScore PRE	AScore PRE	0.95
AScore POST	TUGT POST	−0.79	ARMSE PRE	AScore PRE	−0.92
PROM POST	TUGT POST	−0.80	GFA POST	AROM PRE	0.76
RRMSE POST	T10m POST	0.90	RRMSE POST	TUGT PRE	0.67
RScore POST	T10m POST	−0.85	RScore POST	TUGT PRE	−0.70
AScore POST	T10m POST	−0.78	PROM POST	TUGT PRE	−0.86
TUGT POST	T10m POST	0.94	TUGT POST	TUGT PRE	0.86
RRMSE POST	T2min POST	−0.74	T10m POST	TUGT PRE	0.72
RScore POST	T2min POST	0.77	T2min POST	TUGT PRE	−0.85
AScore POST	T2min POST	0.67	RScore PRE	TUGT PRE	−0.88
PROM POST	T2min POST	0.78	ARMSE PRE	TUGT PRE	0.74
TUGT POST	T2min POST	−0.96	AScore PRE	TUGT PRE	−0.84
T10m POST	T2min POST	−0.88	TUGT POST	T10m PRE	0.75
GEA POST	GEFP POST	0.78	T10m POST	T10m PRE	0.78
GFA POST	GFFP POST	0.82	T2min POST	T10m PRE	−0.78
RRMSE POST	RRMSE PRE	0.72	RRMSE PRE	T10m PRE	0.75
TUGT POST	RRMSE PRE	0.79	RScore PRE	T10m PRE	−0.70
T10m POST	RRMSE PRE	0.91	TUGT PRE	T10m PRE	0.78
T2min POST	RRMSE PRE	−0.80	GEA POST	GEA PRE	0.84
RRMSE POST	RScore PRE	−0.82	GEFP POST	GEA PRE	0.73
RScore POST	RScore PRE	0.84	GEA POST	GEFP PRE	0.76
AScore POST	RScore PRE	0.77	GEFP POST	GEFP PRE	0.76
PROM POST	RScore PRE	0.83	GEA PRE	GEFP PRE	0.75
TUGT POST	RScore PRE	−0.97	RRMSE POST	GFA PRE	0.75
T10m POST	RScore PRE	−0.90	RScore POST	GFA PRE	−0.77
T2min POST	RScore PRE	0.95	AScore POST	GFA PRE	−0.82
RRMSE PRE	RScore PRE	−0.77	PROM POST	GFA PRE	−0.69
RRMSE POST	ARMSE PRE	0.72	TUGT POST	GFA PRE	0.72
RScore POST	ARMSE PRE	−0.68	RScore PRE	GFA PRE	−0.71
AScore POST	ARMSE PRE	−0.72	AScore PRE	GFA PRE	−0.74
TUGT POST	ARMSE PRE	0.93	RScore POST	GFFP PRE	−0.71
T10m POST	ARMSE PRE	0.85	PROM POST	GFFP PRE	−0.84
T2min POST	ARMSE PRE	−0.95	TUGT POST	GFFP PRE	0.69
LMBT POST	ARMSE PRE	−0.78	RScore PRE	GFFP PRE	−0.76
RRMSE PRE	ARMSE PRE	0.78	AScore PRE	GFFP PRE	−0.75
RScore PRE	ARMSE PRE	−0.91	TUGT PRE	GFFP PRE	0.71
RRMSE POST	AScore PRE	−0.80	GFA PRE	GFFP PRE	0.83

RRMSE, resistive root mean squared error; RScore, resistive score; AScore, active score; PROM, passive range of motion; ARMSE, active root mean squared error; TUGT, timed up and go test; T10m, 10 m walking test; T2min, 2 min walking test; GFA, goniometry flexion A; AROM, active range of motion; GEA, goniometry extension A; GEFP, goniometry extension FP; GFFP, goniometry flexion FP; LMBT, left monopodal balance test.

## 5 Discussion

Among the exoskeleton prototypes tested for ankle dysfunctions, the present study is a novel local study in Mendoza (Argentina) that investigates the effects of passive stretching combined with active and resisted ankle movement. It also investigates the impact of passive stretching on gait using the MEXO robotic exoskeleton in patients with stroke sequelae.

Patients received the therapy for 6 weeks, accompanying conventional treatment. Pre- and post-treatment results were compared for the studied variables. There were three variables that showed a net increase at the end of the treatment: kinematics (goniometry), balance through the monopodal test, and distance covered through the 2-min walking test. Muscle tone, the 10-m walking test, and the Timed Up and Go test showed no significant changes. In goniometry, in active flexion, the improvement was not significant, but it was significant in Forced Passive flexion and Forced Passive and Active extension. Calderón Bernal et al. (2015) reported that human gait is a repetitive functional movement, so that therapy, as in the case of the exoskeleton, should be intensive and aimed at stimulating central pattern generators. They also stated that treatment sessions could improve ankle motor skills explained by an increase in cortical motor excitability for the tibialis anterior, thus achieving an increase in dorsi–plantarflexion of the ankle. Several previous studies with other devices reported improvements in passive joint range, muscle strength, and walking speed (Forrester et al., 2011; Zhou et al., 2015; Chang et al., 2017). The walking speed obtained indirectly, taking into account the distance covered in a 2–minute walking test, was 1.07 m/s pre–treatment and 1.20 m/s post–treatment, thus presenting an improvement of 0.13 m/s. According to Duncan et al. (2011), a speed greater than 0.8 m/s is considered to be high. Other authors, such as Chang et al. (2017) and Forrester et al. (2011), found mainly an increase in walking speed. Balance also showed statistically significant improvements, in line with other studies using other prototypes, albeit measured by the Berg balance scale (Chang et al., 2017). It can be observed that the MEXO exoskeleton allows the therapists’ action to be alleviated, increasing the quality of the sessions, their repeatability, length, intensity, and the number of repetitions; as well as the objectivity in the measurement of variables; as robotic devices, machines they are, provide therapists the capability to exactly repeat precise movements, as well as objectively measured, *via* embedded or parallel sensors, physical variables than can be correlated to clinical scales. If these data imply an improvement in ankle control, the variation occurs along with that of the clinical scales used to assess improvement after treatment. Consequently, we can consider that these changes in kinematics, balance and covered distance tend to improve similarly to the clinical scales.

The MEXO exoskeleton would be a prototype, according to the classification of Calderón Bernal et al. (2015), of the static and end–effector type, centred on a single joint. In this case aided by gamification. All patients were able to use the visual feedback with their limbs having a kinematic and dynamic interaction with the robot generating a closed loop between the brain and the limb, and between the human and the robot. Active participation during treatment may improve patients’ adherence and initiative and make them more interested (Chan. 2002; Zhou et al., 2015; Gui et al., 2017). The real effect of video games may be to improve the ability to learn new tasks by increasing attentional control in the cognitive interaction of the human–virtual environment (Green and Bavelier, 2012; Asín-Prieto et al., 2020).

Similarly to what we found in our previous study that applied a haptic adaptive feedback (HAF) adapted to the user capabilities with a more complex (yet similarly designed) tool (Asín-Prieto et al., 2020), the patients improved their range of motion and clinical scales score (for T10m and TUGT). In the HAF study, we also gathered the performance data within the game task, and correlated it with the clinical scales, finding that an improvement in the former could be correlated with better scales in the latter. In the MEXO study we have also used the visual interface (game) as a means of promoting the adherence to the treatment by the patients, and have gathered the performance data, finding moderate to strong correlations between some clinical scales and tests (T10m, TUGT, Monopodal balance) and robotic-videogame system intrinsic measurements (active ROM, resistive RMSE, active and resistive scores). The MEXO study uses the MEXO tool as a simplification towards a low–cost marketable tool based on the tool used in the HAF study.

As referred to by Hussain et al. in terms of the clinical intervention of robots of this type, MEXO allows the patient to practice part of the task (like end–effector robots), perform many repetitions of a movement, which will promote neuroplastic underlying recovery; and maintain and even increase range of motion by mobilising active joints and muscles in different parts of their full range of motion (Hussain et al., 2021).

MEXO, like other robotic ankle exoskeletons, and as reported by Miao et al. (2018), has an optimal design for ankle rehabilitation, through the performance of stretching exercises for the treatment of foot drop, which are usually performed along the dorsiflexion of the ankle, where patients often have difficulty lifting their toes correctly when walking.

Patient satisfaction measured by the QUEST survey was very satisfactory for all patients. The three most important items for the patient were Effectiveness, Comfort and Ease of use. Patient reactions to the training were very positive.

## 6 Conclusion

Preliminary results in this sample of nine patients have shown safety, usability and patient satisfaction with the use of the exoskeleton.

We aimed at exploring the validity of the combined robotic ankle exoskeleton with a video game, designed to enhance the adherence in the therapy protocol. We approached this objective by providing an appealing and improved visual interface (a video game) while asking them to follow a trajectory depicted as a sequence of collectible onscreen items (resistive and active modes).

Although our actual sample size was small, we found for the studied population that patients obtained positive changes in terms of goniometry and balance, rendering our approach valid and usable for training in stroke survivors. Furthermore, the QUEST Likert–like questionnaire showed that the approach of using the integrated MEXO device was very positively received by all patients, with an overall score of 5 over 5; being the three most important aspects (selected by more than half of the sample), from least to most: Comfort, Effectiveness and Ease of Use.

The MEXO device has been shown to be safe during the execution of this study, with no type of inconvenience arising with the patients. It has an easily accessible cut–off switch and so far it has never been necessary to use it. No adverse effects or inconveniences have arisen during the use by patients. In conclusion, the integrated tool including non–ambulatory robotic ankle foot orthosis and video game, together with the delivered treatment protocol has been very positively received by patient end-users, and has led to positive results in terms of improvement in clinical scales, balance and range of motion.

### 6.1 Limitations of this study

The sample size can be considered a limitation of this preliminary exploratory study. All subjects received the same treatment, thus we can obtain conclusions on usability of the system and subjective perception of the end users. However, a randomized controlled trial will be needed to verify the impact of the MEXO robot.

We gathered several representative data, including muscle strength and tone, from a clinical scale point of view. It would have been also useful to provide an objective force measure (torque at the instrumented ankle joint) to compare and even correlate with the clinical scales. Two of the 9 actual participants needed technical aids to walk (in particular, those with a longer time from the lesion). It would be useful to broaden the studied population to survivors with worse walking ability, to try to extend the results to a wider sample of the stroke survivors.

## Data Availability

The raw data supporting the conclusion of this article will be made available by the authors, without undue reservation.
